# S-Scheme 2D/2D Heterojunction of ZnTiO_3_ Nanosheets/Bi_2_WO_6_ Nanosheets with Enhanced Photoelectrocatalytic Activity for Phenol Wastewater under Visible Light

**DOI:** 10.3390/molecules28083495

**Published:** 2023-04-15

**Authors:** Cheng Zuo, Xishi Tai, Zaiyong Jiang, Meifang Liu, Jinhe Jiang, Qian Su, Xueyuan Yan

**Affiliations:** College of Chemistry & Chemical and Environmental Engineering, Weifang University, Weifang 261000, China

**Keywords:** 2D/2D nanosheet-like ZnTiO_3_/Bi_2_WO_6_, S-scheme heterojunction, phenol wastewater pollution, photoelectrocatalytic degradation

## Abstract

The pollution of phenol wastewater is becoming worse. In this paper, a 2D/2D nanosheet-like ZnTiO_3_/Bi_2_WO_6_ S-Scheme heterojunction was synthesized for the first time through a two-step calcination method and a hydrothermal method. In order to improve the separation efficiency of photogenerated carriers, the S-Scheme heterojunction charge-transfer path was designed and constructed, the photoelectrocatalytic effect of the applied electric field was utilized, and the photoelectric coupling catalytic degradation performance was greatly enhanced. When the applied voltage was +0.5 V, the ZnTiO_3_/Bi_2_WO_6_ molar ratio of 1.5:1 had highest degradation rate under visible light: the degradation rate was 93%, and the kinetic rate was 3.6 times higher than that of pure Bi_2_WO_6_. Moreover, the stability of the composite photoelectrocatalyst was excellent: the photoelectrocatalytic degradation rate of the photoelectrocatalyst remained above 90% after five cycles. In addition, through electrochemical analysis, XRD, XPS, TEM, radical trapping experiments, and valence band spectroscopy, we found that the S-scheme heterojunction was constructed between the two semiconductors, which effectively retained the redox ability of the two semiconductors. This provides new insights for the construction of a two-component direct S-scheme heterojunction as well as a feasible new solution for the treatment of phenol wastewater pollution.

## 1. Introduction

With the development of the phenol industry, the pollution of phenol wastewater has become increasingly serious [1,2]. Traditional phenol wastewater treatment technology easily causes secondary pollution, which limits its application. The emerging photoelectric catalysis technology is valued by the majority of people because of its conformity to the development concept of green and environmental protection [3,4,5]. However, photocatalysts used in phenol wastewater have many problems. For example, TiO_2_, ZnO, and other materials cannot effectively use sunlight due to their wide band gap and poor response to visible light [6,7,8]. In addition, materials such as CdS and Cu_2_O are unstable due to their own chemical properties and are easily affected by light corrosion [9,10]. These reasons all limit the application of photocatalytic technology in the degradation of phenol wastewater. Therefore, it is urgent to develop new photocatalytic materials with high visible light response and stable chemical properties suitable for the degradation of phenol wastewater.

Recently, Bi_2_WO_6_ has become a research focus because of its layered structure and stable properties. Studies have found that Bi_2_WO_6_ has an excellent valence band structure, but the conduction band cannot reduce oxygen to superoxide radicals and the photogenerated carriers are easy to recombine, which reduces the photocatalytic efficiency [11]. For this reason, it is necessary to modify Bi_2_WO_6_. To address the problem of photogenerated carrier transfer in traditional heterojunction and Z-scheme heterojunction photocatalysts, Fu et al. proposed the concept of S-scheme (Step-scheme) heterojunction design based on Z-scheme heterojunction without introducing electronic media (redox ion pairs or solid conductors) [12]. The S-scheme heterojunction photocatalyst consists of an oxidation scheme photocatalyst (OP) and a reduction scheme photocatalyst (RP) [13,14]. RP has a higher conduction band, valence band position, and Fermi energy level compared to OP. When RP and OP come into contact, electrons spontaneously transfer from RP to OP until the Fermi energy levels of both reach equilibrium due to the Fermi energy level difference. At this point, a great deal of electrons accumulate at the OP interface, resulting in the energy band bending downward and becoming negatively charged. As electrons are lost at the RP interface, the energy band bends upward and carries a positive charge, eventually forming a built-in electric field from RP to OP [15,16]. After illumination, OP and RP are simultaneously excited to generate photogenerated electrons. Under the synergistic effect of the internal electric field, Coulombic gravity, and band bending, the photogenerated electrons in the OP conduction band are transferred to the RP valence band. Finally, the photogenerated electrons in the RP conduction band and the photogenerated holes in the OP valence band are retained [17,18]. According to the design concept, photogenerated electrons with a weak reduction ability and photogenerated holes with a weak oxidation ability are recombined in S-scheme heterostructures. This improves the separation efficiency of photogenerated electrons and holes, retains photogenerated holes with a strong oxidation ability, and photogenerated electrons with a strong reduction ability, thereby enhancing the oxidation and reduction ability of the catalytic system [19,20]. Bi_2_WO_6_ is an excellent oxidized semiconductor due to its strong oxidation capacity of photogenic holes due to the advantage of valence band. The latest research shows that the usage of Bi_2_WO_6_ to construct S-scheme heterojunction has an excellent effect in the photocatalytic degradation of pollutants. Li et al. designed and constructed an S-scheme Bi_2_WO_6_/CoIn_2_S_4_ heterojunction, which greatly enhanced the TC degradation effect [21]. Therefore, it is still a huge challenge to directly construct a two-component S-scheme heterojunction without introducing expensive electron-mediated materials.

In addition, the latest research shows that the conduction band structure of ZnTiO_3_ is excellent and that the conduction band potential can reduce oxygen to superoxide radicals [22]. Researchers have used ZnTiO_3_ in the field of photocatalysis and have achieved certain results. Chen et al. found that the ZnTiO_3_/Zn_2_Ti_3_O_8_/ZnO ternary photocatalyst had an excellent performance in degrading organic pollutants due to its unique S-scheme heterostructure [23]. Lu et al. found that zinc titanate-based heterostructures had an enhanced photocatalytic performance [24]. The ZnTiO_3_/Zn_2_Ti_3_O_8_/ZnO heterojunction prepared via a simple phase-change method had an excellent performance in hydrogen production and water pollution treatment [25]. According to previous studies, we can see the superiority of the conduction band performance of ZnTiO_3_. Therefore, ZnTiO_3_ is an excellent reducing semiconductor. Therefore, the recombination by coupling ZnTiO_3_ with a high-light electron-generating reduction ability and Bi_2_WO_6_ with a high-light hole-generating oxidation ability at the interface to construct an S-scheme heterojunction is expected. The recombination with ZnTiO_3_ will definitely make up for the conduction band potential of Bi_2_WO_6_ not forming •O_2_^−^, and the interlacing of the two semiconductor band structures can well separate photogenerated electrons and holes. Finally, excellent results have been achieved in the degradation of phenolic pollutants.

The morphologies of the catalysts are varied. Among them, the nanosheet catalyst is more advantageous because the short electron–hole transport path reduces the loss in the transport process and the sheet structure can provide more photoactive sites for photocatalysis to promote the occurrence of the photocatalytic reaction. At present, there are no reports on the work of the S-scheme heterojunction of ZnTiO_3_/Bi_2_WO_6_ in the photocatalytic degradation of phenol wastewater. In this work, a 2D/2D nanosheet ZnTiO_3_/Bi_2_WO_6_ heterojunction system was constructed through the combination of calcining and a hydrothermal method. In addition, the photogenerated carrier was easily recombined during the process of transferring to the catalyst surface for reaction. In order to further improve the separation efficiency of photogenerated carrier, the photoelectric coupling effect of an applied electric field was an effective strategy. The photoelectrochemical performance and photocatalytic performance of the catalyst were studied, and the electron–hole transfer mechanism and functional groups of the photocatalyst were explored. Excellent results were achieved in terms of similar pollutants. This work provides new insights for the construction of a two-component S-scheme heterojunction as well as a feasible new solution for the treatment of phenol wastewater pollution.

## 2. Results and Discussion

### 2.1. XRD Analysis

**Figure 1:**
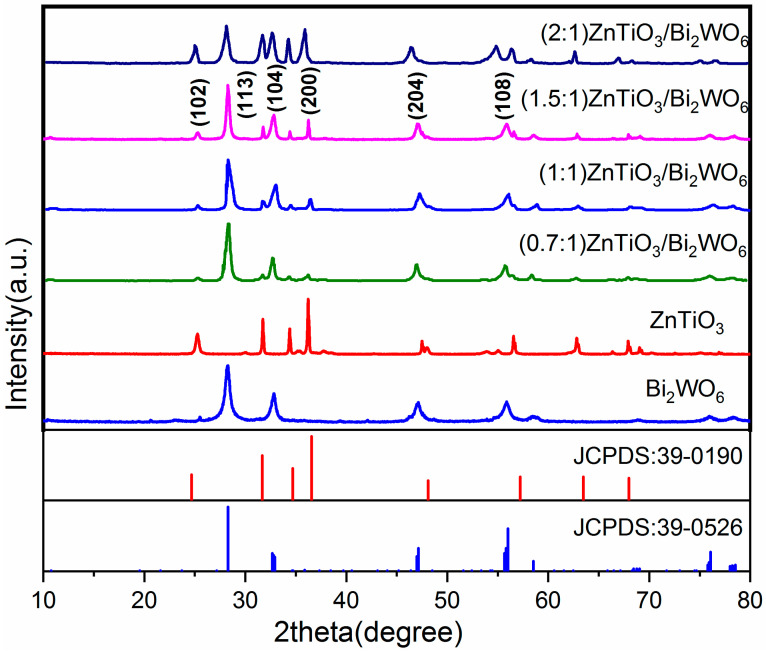
XRD patterns of pure ZnTiO_3_, pure Bi_2_WO_6_, and the ZnTiO_3_/Bi_2_WO_6_ heterojunction.

The crystal structure and crystal plane of the sample were characterized by using XRD. Figure 1 shows that the peaks at 28.2°, 32.9°, 47.2°, 56.0°, and 58.5° corresponded to (131), (200), (220), (133), and (107) of Bi_2_WO_6_, respectively, according to JCPDS card No. 39-0256. On the crystal plane, compared with the standard card, there was no miscellaneous peak, indicating that the prepared Bi_2_WO_6_ had no impurities such as Bi_2_O_3_ or WO_3_ [26]. Figure 1 shows the characteristic peaks of the hexagonal phase ZnTiO_3_ after hydrothermal calcination. According to JCPDS card No. 39-0190, the diffraction angles of 24.1°, 32.8°, 35.3°, 48.8°, 56.8°, 63.3°, and 68.5° corresponded to the (102), (104), (110), (204), (108), (300), and (208) crystal planes of ZnTiO_3_, respectively. No impurity peaks such as TiO_2_ were found, indicating that there was no TiO_2_ in the prepared ZnTiO_3_. The diffraction peaks after the two composites became sharp and narrow, indicating that the particle size of the composite catalyst became larger, so the stronger diffraction peaks could be attributed to the increase in the crystallinity of the composite catalyst [27]. In terms of the number of peaks, the diffraction peak of the composite catalyst corresponded to the peak of the single catalyst, indicating that the two semiconductors had successfully combined.

### 2.2. Morphology Analysis

**Figure 2:**
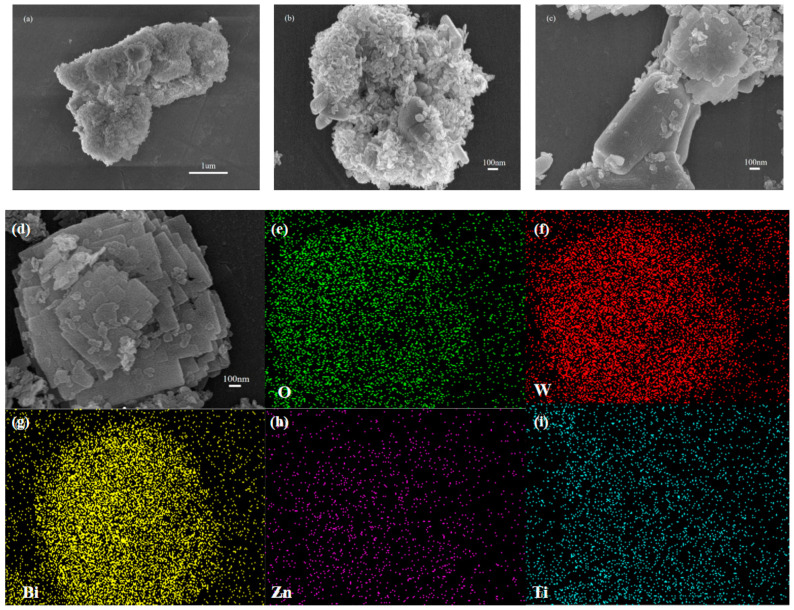
(**a**,**b**) SEM images of the ZnTiO_3_ sample; (**c**,**d**) SEM images of the ZnTiO_3_/Bi_2_WO_6_ heterojunction sample inset in (**d**): EDX of the ZnTiO_3_/Bi_2_WO_6_ heterojunction sample; and the elemental O (**e**), W (**f**), Bi (**g**), Zn (**h**), and Ti (**i**) mapping of the ZnTiO_3_/Bi_2_WO_6_ heterojunction sample.

The morphology of the prepared catalyst can be observed in the SEM images In Figure 2. Figure 2a,b show that the ZnTiO_3_ was a nanosheet cluster composed of small nanosheets; Figure 2c,d show that the ZnTiO_3_/Bi_2_WO_6_ was composed of large nanosheets attached to small nanosheets. The tightly combined structure of small nanosheets and large nanosheets made the two semiconductors in the ZnTiO_3_/Bi_2_WO_6_ composite catalyst come into close contact to construct countless micro-heterojunctions and improve the utilization of visible light. Figure 2e–i show that the O, W, Bi, Zn, and Ti, respectively, were uniformly distributed, and the overall morphology of the large nanosheets of Bi_2_WO_6_ and the cluster shape of the small nanosheets of ZnTiO_3_ can be observed. The larger Bi_2_WO_6_ nanosheet and the smaller ZnTiO_3_ nanosheet were interleaved and in full contact with each other. The 2D nanosheets with unique morphological advantages could effectively shorten the charge-transfer path and provide a platform for the construction of heterogeneous structures. In order to determine the content of each element in the heterojunction catalyst, EDX tests were carried out; the results are shown in the inset in Figure 2d and in Table 1. The atomic ratio of O, W, Bi, Zn, and Ti was 90.12:2.92:4.93:0.62:1.41.

**Figure 3:**
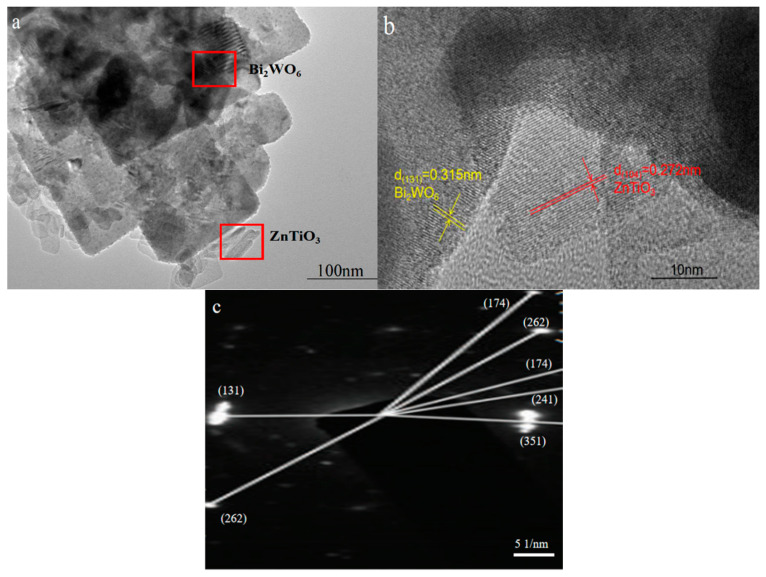
(**a**) TEM image, (**b**) HRTEM image, and (**c**) SAED pattern of the ZnTiO_3_/Bi_2_WO_6_ heterojunction sample.

The structure of the sample as shown in the TEM image could not only be analyzed, but the sample also could be qualitative. It can be seen in Figure 3a that the sample was composed of a large square nanosheet connected to small nanosheets, and this morphology was consistent with that observed in the SEM image. It can be seen in Figure 3b that the large nanosheet with a lattice spacing of 0.315 nm corresponded to the spacing of the Bi_2_WO_6_ (131) crystal plane [28]. The small nanosheet with a lattice spacing of 0.272 nm was consistent with the (104) crystal plane of ZnTiO_3_ [29]. It can be seen in Figure 3a,b that the connection of the two sheet-like structures could construct a heterojunction to inhibit the recombination of photogenerated carriers, and only photogenerated holes and electrons could play a redox effect to generate active groups for the photocatalytic degradation process [30]. In addition, the sheet-like structure could shorten the electron–hole transport path, which allowed holes and electrons to quickly reach the surface of the catalyst, and a redox reaction occurred with the H_2_O and O_2_ attached to the surface, which activated them into the strong oxidizing free radicals •OH and •O_2_^−^. At the same time, the sheet-like structure could provide a large surface area so that the organic pollutant molecules and the strong oxidizing free radicals on the surface of the catalyst were oxidized and decomposed into small inorganic molecules [31]. The SAED pattern of the synthesized heterojunction sample is shown in Figure 3c; the crystal patterns of Bi_2_WO_6_ were mainly (131), (262), (174), (241), and (351). Among these, (131) was the main exposed crystal plane, which was consistent with the XRD results.

### 2.3. XPS Analysis

**Figure 4:**
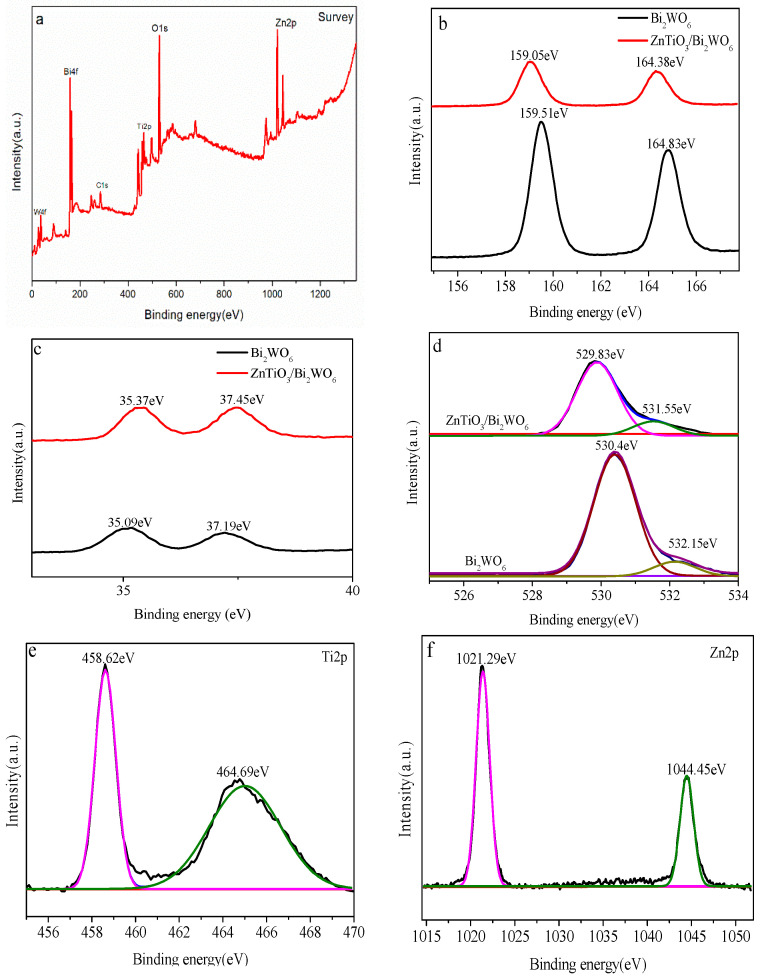
XPS spectra of pure Bi_2_WO_6_ and ZnTiO_3_/Bi_2_WO_6_ heterojunction: (**a**) survey spectrum; (**b**) Bi 4f element spectrum; (**c**) W 4f element spectrum; (**d**) O 1s element spectrum; (**e**) Ti 2p element spectrum; (**f**) Zn 2p element spectrum.

Through XPS characterization, the surface elements and valence state information of the prepared samples were understood. The distribution position of each element can be seen in Figure 4a, indicating that there were Bi, W, O, Ti, and Zn elements in the sample. It can be seen in Figure 4b that 159.05 eV and 164.38 eV corresponded to Bi4f 5/2 and Bi4f 7/2 in the Bi^3+^ valence state, respectively, which was consistent with a previous study [32].

It can be seen in Figure 4c that 35.37 eV and 37.45 eV corresponded to W4f5/2 and W4f7/2 of the W^6+^ valence state, respectively, which was consistent with previous studies [33,34]. In the high-resolution spectrum (Figure 4d) of the O element, the characteristic peak of O1s was decomposed into two peaks, and the binding energies of 529.83 eV and 531.55 eV corresponded to the bond energy of O1s lattice oxygen and surface hydroxyl oxygen, respectively. Two peaks could be fitted in the high-resolution spectrum (Figure 4e) of the Ti element. The binding energies of 458.6 eV and 464.5 eV corresponded to Ti2p3/2 and Ti2p1/2, respectively. The difference between the two peaks was 5.9 eV, indicating that Ti in the sample was Ti^4+^. There were two well-fitted peaks in the high-resolution distribution (Figure 4f). The binding energies of 1021.9 eV and 1045.2 eV corresponded to Zn2p 3/2 and Zn2p 1/2, respectively, indicating that the sample existed in the form of Zn^2+^ [35]. It can be seen in Figure 4b–d that compared with pure Bi_2_WO_6_, the peak of the Bi element in ZnTiO_3_/Bi_2_WO_6_ moved 0.45 eV in the direction of low binding energy, the peak of the W element of ZnTiO_3_/Bi_2_WO_6_ moved 0.28 eV in the direction of high binding energy, and the O element in ZnTiO_3_/Bi_2_WO_6_ moved 0.6 eV in the direction of low binding energy. The shift of binding energy in the XPS spectra was due to the construction of the S-scheme heterojunction between the two semiconductor catalysts. which led to the change in binding energy and the shift in the peak.

### 2.4. Electrochemical Analysis

**Figure 5:**
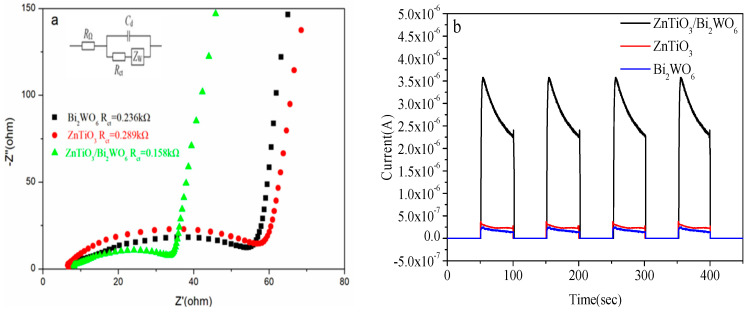
(**a**) Impedance spectrum of the prepared catalyst; (**b**) transient photocurrent curve of the prepared catalyst.

Electrochemical impedance is usually used to detect the rate of charge transfer. The smaller the radius of the arc, the smaller the resistance and the higher the separation efficiency of photogenerated carriers [36]. It can be seen in Figure 5a that the arc radius of ZnTiO_3_/Bi_2_WO_6_ was the smallest. Therefore, the impedance of ZnTiO_3_/Bi_2_WO_6_ was the lowest, the recombination of photogenerated electrons and holes was the smallest, and the photoelectric transmission performance was the best. As shown in Figure 5a, the charge-transfer resistance parameters from the EIS fitting data for the prepared catalysts was tabulated. The charge-transfer resistance parameters of Bi_2_WO_6_, ZnTiO_3_, and Bi_2_WO_6_/ZnTiO_3_ were 0.236 kΩ, 0.289 kΩ, and 0.158 kΩ, respectively. The curvature slope of the impedance curve in the high-frequency range of ZnTiO_3_/Bi_2_WO_6_ was greater than that of Bi_2_WO_6_, indicating that the composite catalyst ZnTiO_3_/Bi_2_WO_6_ had a larger capacitance and better electrochemical properties. Transient photocurrent is usually used to prove the transfer efficiency of a light-excited charge and the stability of photogenerated electrons and holes. As shown in Figure 5b, in the experiment of simulating sunlight with four switches, the photocurrent increased and stabilized instantaneously after illumination, which proved that the photogenerated charge carriers of the photocatalyst were relatively stable. The photocurrent was generated by the light-excited photocatalyst to generate electrons and air. Holes and electrons moved through the ITO glass to transmit current to produce a photocurrent curve. The photocurrent intensity of ZnTiO_3_/Bi_2_WO_6_ was 14 times and 12 times that of pure Bi_2_WO_6_ and pure ZnTiO_3_, respectively, indicating that the composite catalyst had a stronger electron transport capacity than a single catalyst, which further proved that the composite photocatalyst had a high electron–hole separation rate. This indicated that the construction of the S-scheme heterojunction accelerated the separation of charge holes and allowed more charges to work to generate a photocurrent. This result was consistent with the previous electrochemical impedance results.

### 2.5. Photocatalytic Degradation of Phenolic Pollutants

In order to study the performance of the prepared 2D/2D ZnTiO_3_/Bi_2_WO_6_ catalyst, a photoelectrocatalyst degradation experiment was conducted, and the results are shown in Figure 5. When the applied voltage was +0.5 V, there was no electrocatalytic degradation of all kinds of phenolic wastewater in no light, which indicated that the voltage of +0.5 V could not cause electrocatalytic degradation of phenolic wastewater. Therefore, the effect of the applied electric field of +0.5 V was mainly photoelectric coupling, thereby promoting the effective separation of photogenerated electrons and holes. According to Figure 5a and Table 2, by comparing the degradation effects of ZnTiO_3_/Bi_2_WO_6_ with different ratios under the same conditions, we concluded that the molar ratio of 1.5:1 had the best degradation effect; the degradation rate reached 93%. Compared with the pure-phase Bi_2_WO_6_ and ZnTiO_3_ catalysts, the degradation kinetic rate of the composite catalyst for phenol degradation increased by 3.72 times and 2.06 times, respectively. It can be seen in Figure 5b and Table 1 that the composite catalyst with a molar ratio of 1.5:1 had the largest kinetic constant. Compared with the pure Bi_2_WO_6_ and ZnTiO_3_ catalysts, the kinetics of the composite catalyst for degradation of phenol were increased by 3.6 times and 10.42 times, respectively. Compared with the literature, the photocatalytic degradation results in this paper were excellent. The results are shown in Table 3.

**Figure 6:**
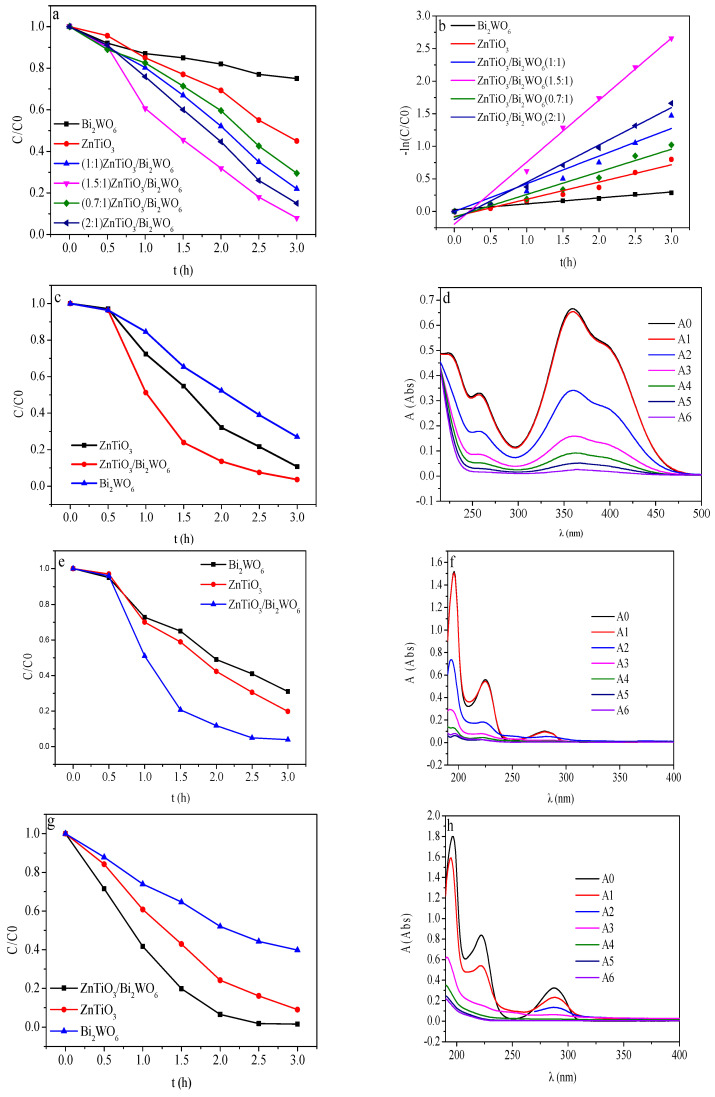
(**a**) Phenol degradation effect and (**b**) kinetic curve of phenol degradation of ZnTiO_3_/Bi_2_WO_6_ with different molar ratios: (**c**) p-nitrophenol, (**e**) p-chlorophenol, and (**g**) p-methylphenol degradation effect by photocatalysts; and full-spectrum UV analysis of ZnTiO_3_/Bi_2_WO_6_ molar ratio of 1.5:1 for (**d**) p-nitrophenol, (**f**) p-chlorophenol, and (**h**) p-methylphenol.

Figure 6a shows the phenol degradation effect. Figure 6b shows the kinetic curves of phenol degradation for different molar ratios of ZnTiO_3_/Bi_2_WO_6_. According to Figure 6c–h, based on the photocatalytic experiments on p-nitrophenol, p-chlorophenol, and p-methylphenol, we found that the degradation efficiency of the photoelectrocatalyst for the methoxy electron-donating group was higher than that for the nitro electron-withdrawing group. This was because the electron-withdrawing groups such as nitro and chlorine groups could form conjugated and stable electrons with a benzene ring and the structure was stronger, and the hyperconjugation effect of electron donating groups caused the benzene ring to become strongly activated and easily reactive, so the characteristic group was easy to separate from the benzene ring, and the strong oxidizing group was more likely to attack the benzene ring and degrade phenols [37,38,39].

**Table 3:** Photocatalytic degradation of phenol wastewater in the presence of semiconductor nanocomposites.

Photocatalyst	Synthesis Methods	Initial Pollutant Conc.	Light Source	Degradation (%)	Ref.
Bi_7_O_9_I_3_/rGO	Solvothermal	10 mg/L	500 W Xe lamp (cutoff filter: λ > 420 nm)	78.3	[40]
Au/BiOBr/graphene	Hydrothermal	10 mg/L	300 W xenon lamp (cutoff filter: λ > 400 nm)	64	[41]
Silica nanosheets (SNSs)—supported mixed phase	Hydrothermal and post-annealing	20 mg/L	300 W Xe lamp with a cut-off filter	90	[42]
GO/SmVO_4_	Sonochemical	1.0 × 10^−4^ mol dm^−3^	35 W LED lamp	90	[43]
TiO_2-x_/g-C_3_N_4_ nanorod arrays	Urea drop-calcined and NaBH_4_ reduction	5 ppm	300 W Xe lamp	87	[44]
g-CN@CuO	Calcination	50 mg/L	500 W Xe lamp with a cut-off filter	87.8	[45]
ZnTiO_3_/Bi_2_WO_6_	Hydrothermal	10 mg/L	350 xenon lamp (λ ≥ 400 nm)	93	This work

In addition, the most easily oxidized site for phenol and its derivatives was located at the ortho position to the phenolic hydroxyl group. The methyl group was located at the para position of the phenolic hydroxyl group as an electron-donating group, making the ortho position more easily oxidized. The nitro and chlorine groups as electron-withdrawing groups had a passivation effect on the benzene ring, which made the ortho position of the phenolic hydroxyl group more difficult to oxidize. The electron-withdrawing ability of nitro group was stronger than that of chlorine group, so it was more difficult to oxidize.

**Figure 7:**
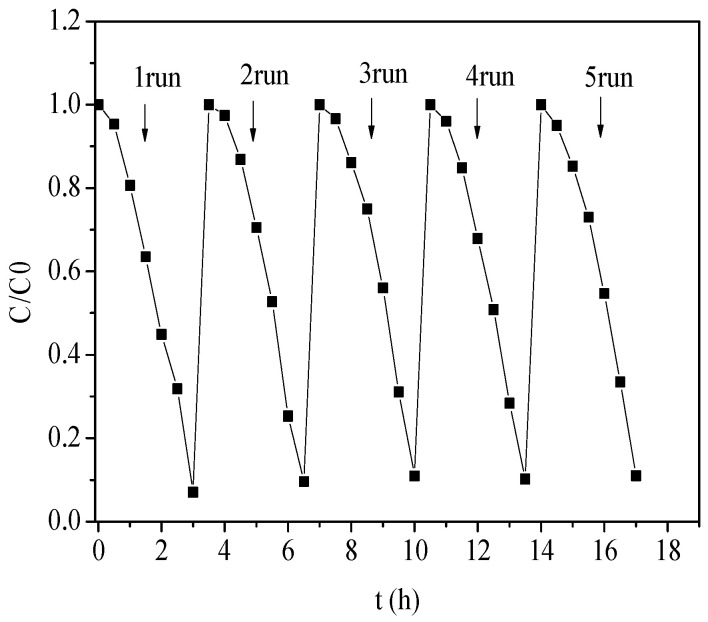
Cyclic stability test curve of ZnTiO_3_/Bi_2_WO_6_.

The stability of the photoelectrocatalytic activity of the recovered catalyst was evaluated via cyclic degradation experiments. It can be seen in Figure 7 that the stability of the composite photoelectrocatalyst was excellent. After five cycles, the photoelectrodegradation effect of the photoelectrocatalyst had hardly changed and was still maintained at more than 90%. The high stability of the photoelectrocatalyst also provided the possibility of industrialization of the photoelectrocatalytic treatment of phenolic wastewater [46].

**Figure 8:**
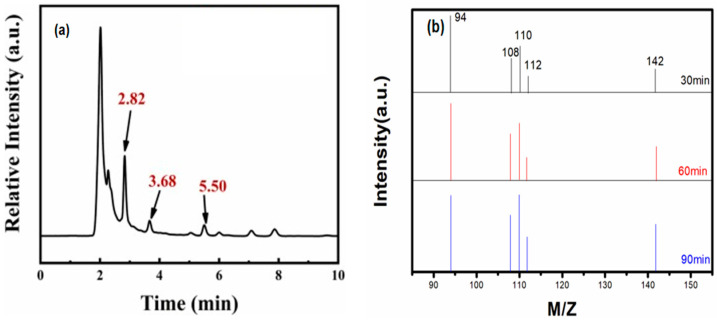
(**a**) Intermediates of phenol degradation and (**b**) mass-to-charge ratio of products after 30, 60, and 90 min of degradation as identified by GC-MS.

Figure 8a shows that the phenol degradation was intermediate. In Figure 8, the HRMS has been provided for the products after 30, 60, and 90 min of degradation. M/Z = 94 represents phenol; M/Z = 110 represents hydroquinone, catechol, and resorcinol; M/Z = 108 represents p-phenyldiquinone, o-phenyldiquinone, and m-phenyldiquinone; and M/Z = 142 represents maleic acid. After the degradation of phenol, many organic products were produced, among which the main intermediate products were hydroquinone, p-benzoquinone, and maleic acid. The content of maleic acid increased with the longer degradation time. These results indicated that the main path of photocatalytic degradation of phenol was phenol → hydroquinone → -p-benzoquinone → maleic acid.

### 2.6. Possible Photoelectrocatalytic Mechanism

**Figure 9:**
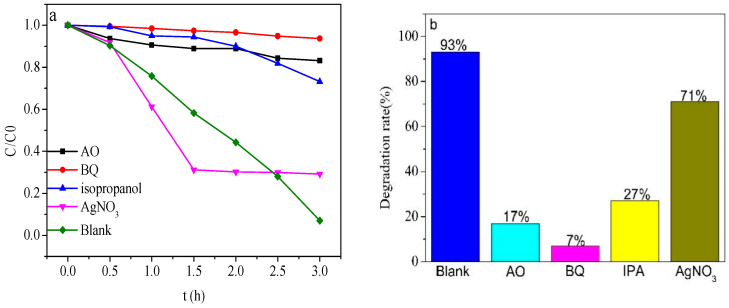

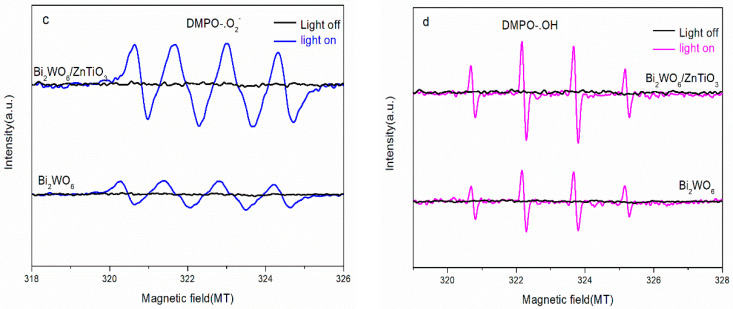
Effect of quencher on phenol degradation rate. (**a**) Degradation curve diagram, (**b**) histogram of degradation rate, (**c**) DMPO-.O_2_^−^, and (**d**) DMPO-.OH spin-trapping ESR spectra for Bi_2_WO_6_ and Bi_2_WO_6_/ZnTiO_3_.

The photoelectrocatalytic mechanism of ZnTiO_3_/Bi_2_WO_6_ under visible light with the applied voltage of +0.5 V was worth pondering. In order to study the mechanism of ZnTiO_3_/Bi_2_WO_6_, free radical-trapping experiments were carried out. We captured •OH with isopropanol(IPA), h^+^ with ammonium oxalate (AO), •O_2_^−^ with 1,4-benzoquinone (BQ), and e^−^ with AgNO_3_. The concentration of IPA, TEOA, BQ, and AgNO_3_ was 1 mM. As shown in Figure 9a,b, after adding BQ and AO, the photocatalytic efficiency decreased from 93% to 7% and 17%, respectively, which indicated that •O_2_^−^ and h^+^ were the main active groups. After adding IPA, the photocatalytic efficiency dropped from 93% to 27%, indicating that •OH was the secondary active group.

After adding AgNO_3_, the photoelectrocatalytic efficiency dropped from 93% to 71%, indicating that e^−^ mainly played a supplementary role and was not an active group. After AgNO_3_ was added, Ag^+^ combined with e^−^ to generate Ag nanoparticles under light conditions, which quenched e^−^. The generated Ag nanoparticles promoted photogenerated electrons and holes due to the local surface plasmon resonance effect when the Ag content was low, which was beneficial to the degradation of phenol, so before 1.5 h, the degradation effect of phenol was better than that of blank. After 1.5 h, the content of Ag nanoparticles was higher than that of the generated Ag nanoparticles, and Ag nanoparticles became new recombination centers of photogenerated electrons and holes, which greatly reduced the degradation effect of phenol.

In addition, it can be seen in the ESR spectra in Figure 9c,d that for Bi_2_WO_6_/ZnTiO_3_, the signal of .OH and the signal of .O_2_^−^ were obviously enhanced, which was consistent with the results of the free radical-capture experiment. This fully proved that the photogenerated carrier separation efficiency of Bi_2_WO_6_/ZnTiO_3_ was significantly improved. The performance of PEC to degrade phenolic wastewater was improved, which was mainly due to the accumulation of h^+^ and .OH in the valence band of Bi_2_WO_6_ and e^−^ and .O_2_^−^ in the conduction band of ZnTiO_3_. This also indirectly proved the structure of the S-scheme heterojunction in Bi_2_WO_6_/ZnTiO_3_.

**Figure 10:**
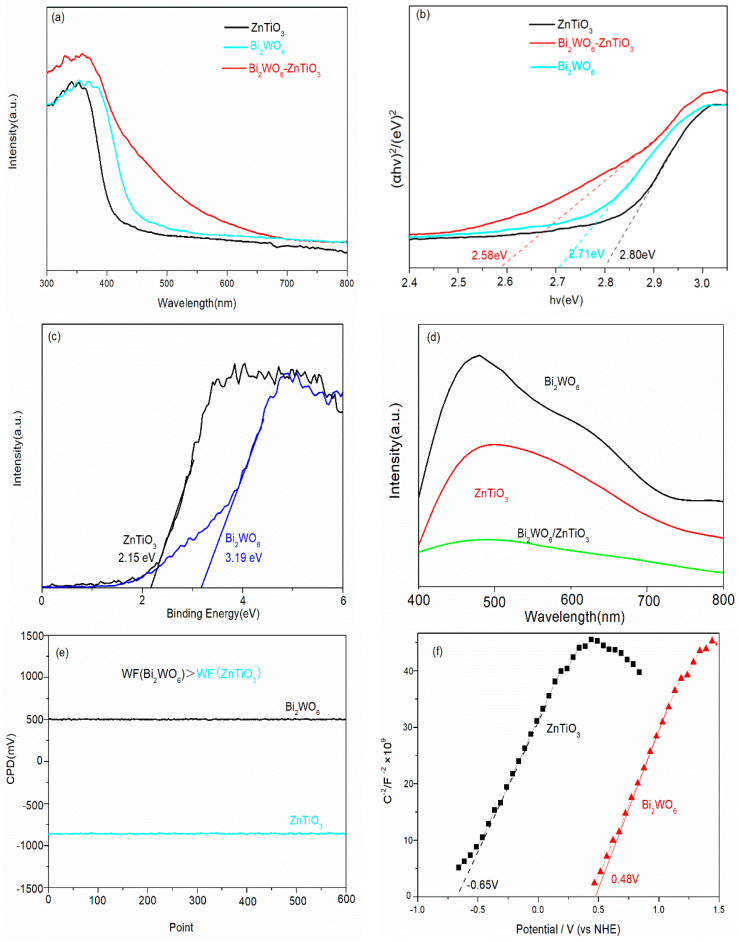
(**a**) UV–vis spectroscopy, (**b**) band gap, (**c**) valence band position, (**d**) PL spectroscopy, and (**e**) work function of the Bi_2_WO_6_, ZnTiO_3_, and Bi_2_WO_6_/ZnTiO_3_ catalysts; and (**f**) the flat band potentials according to a Mott–Schottky electrochemistry spectroscopy test.

In order to determine the band gap, valence band position, photogenerated carrier separation efficiency, and Fermi level of Bi_2_WO_6_, ZnTiO_3_, and Bi_2_WO_6_/ZnTiO_3_, UV–vis spectroscopy, valence band XPS, PL, and work function tests were performed. The results is shown in Figure 10. According to Figure 10a, the visible light absorption capacity of Bi_2_WO_6_/ZnTiO_3_ after composition was obviously enhanced. Figure 8b shows that the bandgaps of Bi_2_WO_6_, ZnTiO_3_, and Bi_2_WO_6_/ZnTiO_3_ were 2.71 eV, 2.80 eV, and 2.58 eV, respectively. Figure 10c shows the valence band positions of Bi_2_WO_6_ and ZnTiO_3_ at 3.19 eV and 2.15 eV, respectively. Figure 10d shows that the PL spectral signal of Bi_2_WO_6_/ZnTiO_3_ after composition was significantly weakened and that there was no obvious absorption peak, which indicated that the separation efficiency of the photogenerated carrier was significantly improved. The charge-transfer mechanism at the interface of Bi_2_WO_6_/ZnTiO_3_ after recombination may have been the S-scheme heterojunction.

In order to determine the transport direction of photogenerated electrons and holes in the Bi_2_WO_6_/ZnTiO_3_ catalysts, the work functions of Bi_2_WO_6_ and ZnTiO_3_ were measured using a Kelvin probe system (SKP5050, KP Technology Ltd.). The formula was as follows: WF (Sample) = WF (tip) + CPD. Calibration of the WF (tip) was realized with a standard gold sheet (gold, 5.10 eV). CPD was the contact potential difference between the sample and the tip (gold, 5.10 eV). The results are shown in Figure 10e. The work function of Bi_2_WO_6_ was 5.60 eV and that of ZnTiO_3_ was 4.25 eV. Therefore, the Fermi energy level of the work function of Bi_2_WO_6_ was significantly lower than that of ZnTiO_3_. When ZnTiO_3_ was in contact with Bi_2_WO_6_, photogenerated electrons transferred from the conduction band of Bi_2_WO_6_ to the valence band of ZnTiO_3_ for quenching. In addition, the flat band potentials of the photocatalysts is reported according to the Mott–Schottky test. As shown in Figure 10f, the flat band potential of ZnTiO_3_ was −0.65 V, and the flat band potential of Bi_2_WO_6_ was 0.48 V. Photogenerated holes accumulated in the valence band of Bi_2_WO_6_ with a stronger oxidation capacity, and photogenerated electrons accumulated in the conduction band of ZnTiO_3_ with a stronger reduction capacity, forming an S-scheme heterojunction charge-transfer mechanism at the interface.

**Figure 11:**
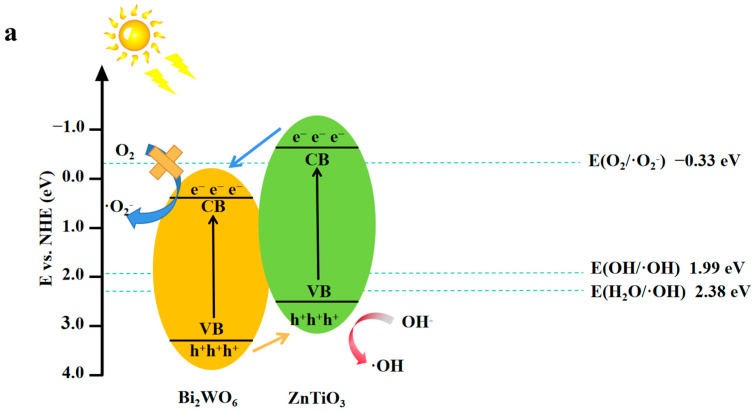

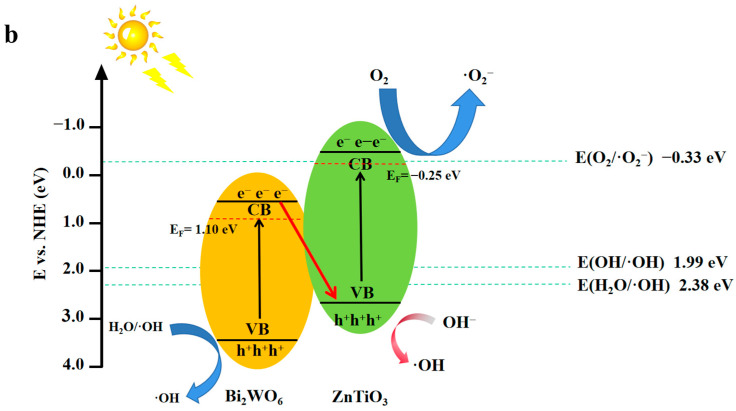
(**a**) P-n heterojunction and (**b**) S-scheme heterojunction mechanism of ZnTiO_3_/Bi_2_WO_6_.

According to the experimental and characterization results, Figure 11 shows the electron–hole transfer pathway and the photoelectrocatalytic mechanism on the ZnTiO_3_/Bi_2_WO_6_ heterojunction. Under visible light irradiation, electrons were excited from the valence band (VB) of Bi_2_WO_6_ and ZnTiO_3_ to the conduction band (CB). Assuming the photocatalytic mechanism was as shown in Figure 11a, because the VB position of Bi_2_WO_6_ was higher than the HOMO position of ZnTiO_3_, the valence band holes of Bi_2_WO_6_ were transferred to the valence band of ZnTiO_3_ because the CB position of Bi_2_WO_6_ was lower than the LUMO position of ZnTiO_3_. The electrons were transferred to the conduction band of Bi_2_WO_6_ [47]. However, because the HOMO potential of ZnTiO_3_ (2.15 eV) was less than E(H_2_O/•OH) (2.38 eV), the amount of holes in the HOMO of ZnTiO_3_ was not enough to oxidize H_2_O to •OH radicals. Therefore, through the reaction, the holes of ZnTiO_3_ could not form·•OH radicals [48]. Since the conduction band potential of Bi_2_WO_6_ was more positive than the standard redox potential of E(O_2_/•O_2_^−^) (−0.33 eV), superoxide radical groups could not be formed, which was inconsistent with the conclusion drawn from the quencher experiment [49]. Assuming that according to the photocatalytic mechanism shown in Figure 8b, the holes of ZnTiO_3_ were combined with the electrons of Bi_2_WO_6_, only the holes of Bi_2_WO_6_ and the electrons of ZnTiO_3_ were retained. Because the lowest unoccupied molecular orbital (LUMO) position of ZnTiO_3_ was more than E(O_2_/•O_2_^−^) (−0.33 eV), the standard redox potential was more negative [50,51]. Therefore, the photoelectrons in ZnTiO_3_ could easily reduce the O_2_ adsorbed on the catalyst surface to generate •O_2_^−^ radicals because the VB position of Bi_2_WO_6_ (3.19 eV) was more correct than the standard redox potentials E(OH^−^/•OH) (1.99 eV) and E(H_2_O/•OH) (2.38 eV). The holes in the VB of Bi_2_WO_6_ reacted with water or OH^−^ in water to form •OH, •OH, and •O_2_^−^ radicals, which oxidatively degraded the phenolic macromolecules into small molecular products. This derivation was consistent with the conclusions of the quencher experiment, so the most likely explanation is that the ZnTiO_3_/Bi_2_WO_6_ heterojunction was a direct S-scheme photoelectrocatalytic mechanism. In the direct S-scheme semiconductor, the heterojunction formed by the two semiconductors not only retained the superior oxidation–reduction potential, but also reduced the recombination rate of photogenerated electron–hole pairs. This solution greatly improved the oxidation–reduction ability of the Bi_2_WO_6_ photoelectrocatalysis.

## 3. Experiments

### 3.1. Materials

Zn(CH_3_COO)_2_ (AR) (99%), Bi(NO_3_)•5H_2_O (AR) (99.999%), Ti(OC_4_H_9_)_4_ (AR) (99%), and Na_2_WO_4_.2H_2_O (AR) (99.99%) were purchased from China Sinopharm Chemical Reagent Co. Ltd., Shanghai, China; ethanol (AR), CO(NH_2_)_2_ (AR) (99.99%), Na_2_SO_4_ (AR) (99.99%), and ethylene glycol (AR) (99.99%) were purchased from Tianjin Kemiou Chemical Reagent Company, Tianjin, China; and the deionized water was made in the laboratory.

### 3.2. Preparation of Bi_2_WO_6_ and ZnTiO_3_

First, 2 mmol of Bi(NO_3_)_3_•5H_2_O and 1 mmol of Na_2_WO_4_•2H_2_O were added to 30 mL of ethylene glycol and ultrasonically dispersed for 10 min. The resulting Na_2_WO_4_•2H_2_O solution was slowly added dropwise to the Bi(NO_3_)_3_•5H_2_O solution and then ultrasonically dispersed for 30 min so the solution was mixed thoroughly. Then, the mixed solution was poured into a 100 mL solvothermal reactor and placed in an oven at 160 °C for heating for 12 h. The prepared product was centrifuged. The lower solid was washed with ethanol and deionized water and dried in a vacuum drying oven at a temperature of 60 °C for 24 h to obtain the prepared Bi_2_WO_6_ powder.

The ZnTiO_3_ with a cluster nanoflake structure was successfully prepared by using a two-step calcination method. First, 8.62 g of Zn(CH_3_COO)_2_ and 9.86 g of Ti(OC_4_H_9_)_4_ were dissolved in a 100 mL ethanol solution of 1 mol/L of urea and stirred evenly, and the solution was poured into 200 mL of solvent. The reaction kettle was placed in an oven at 160 °C and heated for 12 h; the molar ratio of the Zn:Ti elements was (1:1). After washing and drying the obtained ZnTiO_3_ precursor, it was placed into a muffle furnace and calcined at 700° for 3 h and then subjected to secondary heat treatment and calcined at 800° for 5 h to obtain a pure ZnTiO_3_ powder.

### 3.3. Preparation of ZnTiO_3_/Bi_2_WO_6_

The ZnTiO_3_/Bi_2_WO_6_ composite photocatalyst was prepared by using a hydrothermal method. First, 2 mmol of Bi(NO_3_)3.5H_2_O and 1 mmol of Na_2_WO_4_•2H_2_O were each added to 30 mL of deionized water and stirred for 10 min. Then, the Na_2_WO_4_•2H_2_O solution was slowly added dropwise to the Bi(NO_3_)_3_•5H_2_O solution. This mixed solution was called solution A. The pH of solution A was adjusted to 7 with 0.1 mol/L of NaOH solution and stirred for 30 min. According to the different molar ratio of Zn:Bi, the ZnTiO_3_ prepared above was added to 10 mL of deionized water and ultrasonicated for 30 min to make the dispersion uniform. This ultrasonic suspension, which was called solution B, was added to solution A, and the mixed solution was transferred to a 100 mL hydrothermal reactor and kept at 160 °C for 12 h. Then, the prepared product was centrifuged. The lower solid was washed with ethanol and deionized water and dried in a vacuum drying oven at 60 °C for 24 h to obtain ZnTiO_3_/Bi_2_WO_6_ photocatalysts with different molar ratios.

### 3.4. Photoelectrocatalytic Degradation of Phenolic Pollutants

First, 100 mL of 10 mg/L phenol solution, 100 mL of 10 mg/L p-nitrophenol solution, 100 mL of 10 mg/L p-chlorophenol solution, and 100 mL of 10 mg/L 4-methylphenol solution were each selected as a pollutant. The photoelectrocatalytic degradation was carried out with a CHI660E three-electrode system electrochemical workstation. An Ag/AgCl electrode was used as the reference electrode, a Pt electrode was used as the counter electrode, and the photocatalyst-coated ITO conductive glass was used as the working electrode. The preparation process was to first mix 10 mg of catalyst and 5 mL of ethanol solution. The mixed system was ground for 15 min, then a proper amount of supernatant was added to ethanol and diluted for 5 min to uniformity, spin-coated on ITO glass, and dried at 60 °C. The light source was 350 W xenon lamp (λ ≥ 400 nm), the electrolyte solution was a 0.1 mol/L Na_2_SO_4_ solution, and the applied voltage was +0.5 V. Then, photocatalyst-coated ITO and 100 mL of 10 mg/L phenolic pollutant solution was dispersed for 30 min to reach the equilibrium of adsorption and desorption of organic pollutants in the dark. We took 4 mL samples every 30 min under the illumination of the 350 W xenon lamp (λ ≥ 400 nm) to centrifuge and separate the supernatant. We calculated the degradation rate as D = (1 − C_t_/C_0_) × 100%. In the above formula, C_0_ is the concentration of the phenolic solution before degradation, C_t_ is the concentration of the phenolic solution after different degradation times, and D is the calculated degradation rate.

### 3.5. Electrochemical Analysis

The electrochemical analysis was carried out with a CHI660E three-electrode system electrochemical workstation. An Ag/AgCl electrode was used as the reference electrode, a Pt electrode was used as the counter electrode, and a photocatalyst-coated ITO conductive glass was used as the working electrode. The preparation process was to first mix the catalyst and ethanol solution. The mixed system was ground for 15 min, then a proper amount of supernatant was added to ethanol and diluted for 5 min to uniformity, spin-coated on ITO glass, and dried at 60 °C. the light source was a 350 W xenon lamp (λ ≥ 400 nm), and the electrolyte solution was 0.1 mol/L of Na_2_SO_4_ solution. A short photocurrent density measurement was performed during the ON/OFF cycle at 0 V for 450 s. The frequency range test of electrochemical impedance was 100 kW–0.01 W.

### 3.6. Characterization

XRD was measured using an X-ray diffractometer, and CuKα radiation (λ = 1.5418 Å) was performed in the range of 2θ = 10–80°. The scanning electron microscopy (SEM) was performed with a JEOL-1600 field emission microscope with an acceleration voltage of 5 kV. Transmission electron microscopy (TEM) was performed with a JEOL-2100 (Japan JEOL) operating at 200 kV. X-ray photoelectron spectroscopy (XPS) was performed with an ESCALab MKII spectrometer with MgKα radiation, and its binding energy position was calibrated as C 1 s = 284.6 eV. The UV–vis DRS spectra of Bi_2_WO_6_, ZnTiO_3_, and Bi_2_WO_6_/ZnTiO_3_ were recorded with an ultraviolet–visible spectrophotometer (UV 2600).

## 4. Conclusions

1. In this paper, 2D/2D heterojunctions of ZnTiO_3_ nanosheets/Bi_2_WO_6_ nanosheets were prepared for the first time by combining a hydrothermal method and a two-step calcination method, and two types of phenolic pollutants were selected. The effects of photocatalysts on electron-absorbing and electron-donating phenolic pollutants were discussed. It was confirmed that the photocatalyst had an obvious degradation effect on the electron-donating phenolic pollutants. This was because the electron-donating group could accelerate the oxidation of the ortho hydroxyl, making the benzene ring easier to decompose.
2. Compared with pure Bi_2_WO_6_ (25%), the degradation rate of phenol by the ZnTiO_3_/Bi_2_WO_6_ photocatalyst could reach 93%, and the kinetic rate was increased by 3.6 times. The main reasons for the performance improvement were as follows: (1) 2D/2D ZnTiO_3_/Bi_2_WO_6_ heterojunction shortened the charge-transfer path and reduced the resistance of photogenerated electrons and holes to the surface; (2) the S-scheme heterojunction mechanism was constructed at the ZnTiO_3_/Bi_2_WO_6_ interface, which maintained a higher oxidation potential and reduction potential and realized the spatial separation of photogenerated carriers; and (3) the photoelectric coupling effect of the applied electric field further promoted the separation of the photogenerated carrier and improved the active free radical·OH and·O_2_^−^. This work provides a new strategy for the degradation of phenolic wastewater.

## Figures and Tables

**Table 1:** The content of various elements in the Bi_2_WO_6_/ZnTiO_3_ sample according to EDX.

Element	Weight %	Atomic %
O K	46.25	90.12
Ti K	2.16	1.41
Zn K	1.30	0.62
W M	17.20	2.92
Bi M	33.09	4.93
Totals	100.00	100.00

**Table 2:** Kinetic parameters of the prepared catalyst.

Photoelectrocatalysts	Degradation Rate	Slope	R^2^	Kinetic Equation
Bi_2_WO_6_	25%	0.0913	0.986	Y = 0.0913X + 0.02486
ZnTiO_3_	55%	0.264	0.976	Y = 0.264X − 0.07816
ZnTiO_3_/Bi_2_WO_6_(1:1)	77%	0.424	0.989	Y = 0.424X
ZnTiO_3_/Bi_2_WO_6_(1.5:1)	93%	0.9517	0.992	Y = 0.9517X − 0.195
ZnTiO_3_/Bi_2_WO_6_(0.7:1)	71%	0.3472	0.971	Y = 0.3472X − 0.086
ZnTiO_3_/Bi_2_WO_6_(2:1)	81%	0.5733	0.992	Y = 0.5733X − 0.126

## Data Availability

Not applicable.

## References

[1] Antony R.P., Mathews T. (2013). Kinetics of dye destruction using electrochemically synthesized sunlight active N-doped and C and N Co-doped TiO_2_ nanotubular photocatalysts, Energy Environ. Focus.

[2] Ochiai T., Fujishima A. (2012). Photoelectrochemical properties of TiO_2_ photocatalyst and its applications for environmental purification. J. Photochem. Photobiol. C.

[3] Zhang H., Lv X. (2010). P25-Graphene Composite as a High Performance Photocatalyst. ACS Nano.

[4] Zhou W., Fu H. (2013). ChemInform Abstract: Mesoporous TiO_2_: Preparation, Doping, and as a Composite for Photocatalysis. ChemCatChem.

[5] Hou Y., Li X. (2009). Photoeletrocatalytic activity of a Cu_2_O loaded self-organized highly oriented TiO_2_ nanotube array electrode for 4-chlorophenol degradation. Environ. Sci. Technol..

[6] Chen X., Liu L., Yu P.Y., Mao S.S. (2011). Increasing Solar Absorption for Photocatalysis with Black Hydrogenated Titanium Dioxide Nanocrystals. Science.

[7] Luo J.M., Dong G., Zhu Y. (2017). Switching of semiconducting behavior from n-type to p-type induced high photocatalytic NO removal activity in g-C_3_N_4_. Appl. Catal. B Environ..

[8] Deng F., Zhang Q., Yang L. (2018). Visible-light-responsive graphene-functionalized Bi-bridge Z-scheme black BiOCl/Bi_2_O_3_ heterojunction with oxygen vacancy and multiple charge transfer channels for effificient photocatalytic degradation of 2-nitrophenol and industrial wastewater treatment. Appl. Catal. B Environ..

[9] Tada H., Mitsui T., Kiyonaga T., Akita T., Tanaka K. (2006). All-solid-state Z-scheme in CdS-Au-TiO_2_ three-component nanojunction system. Nat. Mater..

[10] Zhu Y., Xu D., Meng M. (2015). Ultrasonic-assisted synthesis of amorphous Bi_2_S_3_ coupled (BiO)_2_CO_3_ catalyst with improved visible light-responsive photocatalytic activity. J. Mater. Sci..

[11] Meng X.C., Li Z.Z., Zeng H.M., Chen J., Zhang Z.S. (2017). MoS_2_ quantum dots-interspersed Bi_2_WO_6_ heterostructures for visible light-induced detoxifification and disinfection. Appl. Catal. B Environ..

[12] Fu J.W., Xu Q.L., Low J.X. (2019). Ultrathin 2D/2D WO_3_/g-C_3_N_4_ step-scheme H_2_-production photocatalyst. Appl. Catal. B Environ..

[13] Bao Y.J., Song S.Q., Yao G.J. (2021). S-Scheme Photocatalytic Systems. Sol. RRL.

[14] Zhang L.Y., Zhang J.J., Yu H.G. (2022). Emerging S-Scheme Photocatalyst. Adv. Mater..

[15] Xu Q.L., Zhang L.Y., Cheng B. (2020). S-Scheme Heterojunction Photocatalyst. Chem.

[16] Wang J., Wang G.H., Cheng B. (2021). Sulfur-doped g-C_3_N_4_/TiO_2_ S-scheme heterojunction photocatalyst for Congo Red photodegradation. Chin. J. Catal..

[17] Chen R.F., Zhou W., Qu W.W., Wang Y.J. (2022). A novel hierarchical nanostructured S-scheme RGO/Bi_2_MoO_6_/Bi_2_WO_6_ heterojunction: Excellent photocatalytic degradation activity for pollutants. Appl. Surf. Sci..

[18] Tang D.Y., Xu D.S., Luo Z.P. (2022). Highly Dispersion Cu_2_O QDs Decorated Bi_2_WO_6_ S-Scheme Heterojunction for Enhanced Photocatalytic Water Oxidation. Nanomaterials.

[19] Li Y.F., Xia Z.L., Yang Q. (2022). Review on g-C_3_N_4_-based S-scheme heterojunction photocatalysts. J. Mater. Sci. Technol..

[20] Han X.X., Lu B.J., Huang X. (2022). Novel p- and n-type S-scheme heterojunction photocatalyst for boosted CO_2_ photoreduction activity. Appl. Catal. B Environ..

[21] Reddy K.H., Martha S., Parida K.M. (2018). Erratic charge transfer dynamics of Au/ZnTiO_3_ nanocomposites under UV and visible light irradiation and their related photocatalytic activities. Nanoscale.

[22] Anwer H., Mahmood A., Lee J. (2019). Photocatalysts for degradation of dyes in industrial effluents: Opportunities and challenges. Nano Res..

[23] Chen F., Yu C., Wei L. (2019). Fabrication and characterization of ZnTiO_3_/Zn_2_Ti_3_O_8_/ZnO ternary photocatalyst for synergetic removal of aqueous organic pollutants and Cr(VI) ions. Sci. Total Environ..

[24] Lu J., Li D., Chai Y. (2019). Rational design and preparation of nanoheterostructures based on zinc titanate for solar-driven photocatalytic conversion of CO_2_ to valuable fuels. Appl. Catal. B-Environ..

[25] Yu C., Chen F., Zhou W. (2019). A facile phase transformation strategy for fabrication of novel Z-scheme ternary heterojunctions with efficient photocatalytic properties. Nanoscale.

[26] Huang J., Tan G., Yang W. (2013). Synthesis and Photocatalytic Properties of N/Bi_2_WO_6_ Flower-like Crystallites Self-Assembled from Nanoflflakes. J. Clust. Sci..

[27] Chi Y., Yuan Q., Hou S., Zhao Z. (2016). Synthesis and characterization of mesoporous ZnTiO_3_ rods via a polyvinylpyrrolidone assisted sol–gel method. Ceram. Int..

[28] Meng X.C., Zhang Z.S. (2017). Synthesis and characterization of plasmonic and magnetically separable Ag/AgCl-Bi_2_WO_6_@ Fe_3_O_4_@SiO_2_ core-shell composites for visible light-induced water detoxification. J. Colloid Interface Sci..

[29] Raveendra R.S., Prashantha P.A., Krishnac R.H. (2014). Synthesis, structural characterization of nano ZnTiO_3_ ceramic: An effective azo dye adsorbent and antibacterial agent. J. Asian Ceram. Soc..

[30] Hu J., Chen D., Mo Z. (2019). Z-Scheme 2D/2D Heterojunction of Black Phosphorus/ Monolayer Bi_2_WO_6_ Nanosheets with Enhanced Photocatalytic Activities. Angew. Chem. Int. Ed..

[31] Xu J., Yue J., Niu J. (2018). Fabrication of Bi_2_WO_6_ quantum dots/ultrathin nanosheets 0D/2D homojunctions with enhanced photocatalytic activity under visible light irradiation. Chin. J. Catal..

[32] Zhu Z.L., Ren Y., Li Q. (2018). One-pot electrodeposition synthesis of Bi_2_WO_6_/graphene composites for photocatalytic applications under visible light irradiation. Ceram. Int..

[33] Wang R.S., Li B., Xiao Y. (2018). Optimizing Pd and Au-Pd decorated Bi_2_WO_6_ ultrathin nanosheets for photocatalytic selective oxidation of aromatic alcohols. J. Catal..

[34] He W., Sun Y., Jiang G. (2018). Activation of amorphous Bi_2_WO_6_ with synchronous Bi metal and Bi_2_O_3_ coupling: Photocatalysis mechanism and reaction pathway. Appl. Catal. B Environ..

[35] Ranjith K.S., Uyar T. (2018). Conscientious Design of Zn-S/Ti N Layer by Transformation of ZnTiO_3_ on Electrospun ZnTiO_3_@TiO_2_ Nanofifibers: Stability and Reusable Photocatalytic Performance under Visible Irradiation. ACS Sustain. Chem. Eng..

[36] Qian W., Greaney P. (2014). Low-Temperature Nitrogen Doping in Ammonia Solution for Production of N-Doped TiO_2_-Hybridized Graphene as a Highly Efficient Photocatalyst for Water Treatment. ACS Sustain. Chem. Eng..

[37] Long G.Y., Ding G.Y. (2018). Fabrication of mediator-free g-C_3_N_4_/Bi_2_WO_6_ Z-scheme with enhanced photocatalytic reduction dechlorination performance of 2,4-DCP. Appl. Surf. Sci..

[38] Zhang Z., Shen Q.H., Cissoko N. (2010). Catalytic dechlorination of 2,4-dichlorophenol by Pd/Fe bimetallic nanoparticles in the presence of humic acid. J. Hazard. Mater..

[39] Arellano-Gonzalez M.A., Texier A.C., Lartundo-Rojas L., Gonzalez I. (2015). Electrochemical dechlorination of 2-chlorophenol on Pd/Ti, Ni/Ti and Pd-Ni Alloy/Ti electrodes. J. Electrochem. Soc..

[40] Gawande S.B., Gawande K.B., Thakare S.R. (2015). Photocatalytic degradation of phenol over novel rod shaped graphene@BiPO4 nanocomposite. J. Phys. Chem. Solids.

[41] Yu X., Wang L., Feng L.J. (2016). Preparation of Au/BiOBr/Graphene composite and its photocatalytic performance in phenol degradation under visible light. J. Fuel. Chem. Tech..

[42] Wang L., Wang X., Yin J. (2016). Silica induced oxygen vacancies in supported mixed-phase TiO_2_ for photocatalytic degradation of phenol under visible light irradiation. Catal. Commun..

[43] Shandilya P., Mittal D., Soni M. (2018). Fabrication of fuorine doped graphene and SmVO4 based dispersed and adsorptive photocatalyst for abatement of phenolic compounds from water and bacterial disinfection. J. Clean Prod..

[44] Qi F., An W.J., Wang H. (2020). Combing oxygen vacancies on TiO_2_ nanorod arrays with g-C3N4 nanosheets for enhancing photoelectrochemical degradation of phenol. Mater. Sci. Semicond. Process.

[45] Baishnisha A., Divakaran K., Balakumar V. (2021). Synthesis of highly efcient g-CN@CuO nanocomposite for photocatalytic degradation of phenol under visible light. J. Alloy Compd..

[46] Chen W., Liu T.Y., Huang T. (2015). In situ fabrication of novel Z-scheme Bi_2_WO_6_ quantum dots/g-C_3_N_4_ ultrathin nanosheets heterostructures with improved photocatalytic activity. Appl. Surf. Sci..

[47] Jiang D.L., Wan X. (2018). Enhanced photocatalytic activity of graphitic carbon nitride/carbon nanotube/Bi_2_WO_6_ ternary Z-scheme heterojunction with carbon nanotube as efficient electron mediator. J. Colloid Interface Sci..

[48] Guo H.P., Hayakawa T., Nogami M., Hao Z. (2015). Zinc titanium glycolate acetate hydrate and its transformation to zinc titanate microrods: Synthesis, characterization and photocatalytic properties. Rsc Adv..

[49] Li X.B., Xiong J., Huang J.T. (2019). Novel g-C_3_N_4_/ h′ ZnTiO_3_-a′ TiO_2_ direct Z-scheme heterojunction with significantly enhanced visible-light photocatalytic activity. J. Alloy. Compd..

[50] Ruan X., Hu Y.Y. (2020). Effectively enhanced photodegradation of Bisphenol A by in-situ g-C_3_N_4_-Zn/Bi_2_WO_6_ heterojunctions and mechanism study. Chemosphere.

[51] Li B.R., Cui Y.H., Feng Y. (2020). Study of enhanced photocatalytic performance mechanisms towards a new binary-Bi heterojunction with spontaneously formed interfacial defects. Appl. Surf. Sci..

